# Early Treatment of Mental Cases: Proposed Use for Reception Homes and Observation Wards

**Published:** 1914-08-01

**Authors:** 


### EARLY TREATMENT OF MENTAL CASES.

### Proposed Use for Reception Homes and Observation Wards.

A deputation of medical men and others interested in the matter waited on Mr. Herbert Samuel, President of the Local Government Board, recently, to advocate the provision of greater facilities for the treatment without certification of incipient mental cases. The deputation submitted a recommendation that there should be more opportunity for the temporary preventive treatment of such cases in reception homes, observation wards of Poor-Law infirmaries, or the wards of general or special hospitals. They suggested that patients should enter such wards as they would enter a hospital—*i.e.* on an absolutely voluntary footing and without certification, authorisation, or compulsion. The deputation laid stress upon the fact that it was most desirable that such wards and homes should not be regarded as half-way houses to "asylums," and that they should therefore be kept wholly outside the jurisdiction of the Board of Control. They considered also that, except in cases of urgency, no patient, who had thus voluntarily entered an observation ward, should be certified except by independent doctors after his discharge from the institution.

Dr. Chapple, speaking in support of the objects of the deputation, referred to the work that had been done in Scotland. He said that out of 1,116 mental patients sent into the observation wards of the Eastern District Hospital in Glasgow less than half had had to go to an asylum. The results had been so satisfactory that he wanted the Local Government Board in England to introduce similar methods in the English Poor-Law infirmaries.

The Scottish system to which reference was thus made began so far back as 1890. In that year the Glasgow Parish Council, with the knowledge and approval of the Lunacy Commissioners, established two small wards of six beds each in the Barnhill Poorhouse for the reception and treatment without certification, for short periods, of cases of incipient insanity. The success attending this experiment was so great that in 1904 the same Council built a small pavilion of fifty beds, twenty-five for each sex, in the grounds of their Eastern District Hospital. These wards are spacious and handsome, and admirably designed and equipped for their purpose. The nursing is performed as in the ordinary wards by a female staff, assisted, however, by male nurses on the male side. Other Parish Councils followed the example of Glasgow, and in 1906 the Scottish Local Government Board, after conferring with the Lunacy Commissioners, in order to prevent any possible abuses or irregularities arising, drew up a memorandum of conditions on which they would be prepared to approve of the establishment or continuance of observation wards in the Scottish poorhouses. The most important of these conditions are the following:

1. Observation wards must be separated from the other wards of the poorhouse. They must resemble hospital wards, and contain at least 1,000 cubic feet of air space per bed. There should be single rooms in proximity to the main ward for those patients whom it is desirable to place by themselves.

2. No inmate who is not medically certified as suitable for treatment in an observation ward is to be lodged therein. No patient is to remain for a longer period than six weeks, except with the consent of the Local Government Board.

3. If the observation wards contain sixteen beds or more there must be at least one resident medical officer.

4. The day nursing staff must consist of at least one nurse to eight patients, and the night staff of one nurse to ten patients. Where female nurses are employed for the nursing of male patients either by day or night, a male official should at all times be available in case of emergency.

With this memorandum the Local Government Board circulated the following letter, which indicates the classes of cases experience has proved to be most likely to receive benefit from treatment of this kind:

The Local Government Board feel that a wide discretion must be left to the medical officers in charge of these wards, and to the medical officers who sign the certificates of admission. No doubt there will sometimes be diversity of opinion as to whether a patient sent to an observation ward should not more properly have been sent to an asylum as a certified lunatic. In such cases the considerations to be kept chiefly in mind are the immediate cause, and the probable duration, of the mental disturbance. As a general rule, it is not intended that cases should be kept in an observation ward longer than six weeks. It is difficult to limit or to specify exactly the type of case for which observation wards are suitable, but the following may be mentioned:

(*a*) Where the mental symptoms are a sequel or accompaniment of diseases that in ordinary circumstances terminate within a definite time. The point specially to be kept in view here is the likelihood of the speedy disappearance of the symptoms of mental disturbance.

(*b*) Where, although the mental symptoms would seem to indicate lunacy, the medical officer is clearly of opinion that such symptoms are likely to be of short duration.

(*c*) Where the patient's mental state gives rise to apprehension, but where the symptoms are not sufficiently marked to enable the certifying physician to affirm either sanity or insanity.

(*d*) Where the mental disorder is associated with alcoholic abuse.

(*e*) Senile cases where there are temporary symptoms of mental derangement which make it undesirable that the patients should be treated in a general hospital ward.

(*f*) The presence of the following conditions should be regarded as contra-indicating suitability for such wards:

1. Homicidal tendencies.
2. Dangerous violence.
3. Acute and persistent suicidal tendencies.
4. Long established insanity or known existence of chronic delusions.

Although the matter has never been systematised in England it is to be noted that the classes enumerated in the paragraphs (a) to (e) of this letter are very often admitted to the ordinary wards of Poor-Law infirmaries and dealt with as ordinary inmates. Cases, too, coming under these headings and brought to the infirmary by the police or relieving officers on a three-day order under Section 20 of the Lunacy Act are often, after being seen by a justice of the peace, transferred to the ordinary wards and treated as ordinary patients. The consequence has been that in England, just as in Scotland, only about 50 or 60 per cent. of the patients sent in as lunatics are transferred to asylums. The difficulties that have been found to exist in practice fall under three headings, two of which received very little or no consideration at the hands of the deputation. In the first place the Lunacy Commissioners always laid stress upon the fact that patients who were certifiable under the Lunacy Acts ought to be dealt with formally and placed under certificates. In the second place, and this is the greatest difficulty of all, many of these patients suffering from the early stages of mental disease will not consent to remain voluntarily in the infirmary for a sufficient length of time. Directly the immediate symptoms of which they complain are ameliorated they are anxious to return to their homes and occupations. They insist upon taking their discharge in face of all advice, with the result that an early relapse occurs only too often. In such cases powers are required to detain the patient in the infirmary for a limited and stated period for purposes of treatment. In the third place the absence of any provision for the treatment of these cases in convalescent homes in the country when they are sufficiently well to be discharged from the infirmary is a great drawback in the case of infirmaries in large towns. If properly equipped convalescent homes were provided—as they could easily be by combinations of Guardians—much of the difficulty referred to under the second heading would at once disappear. The obvious advantages to their general health to be derived from a stay in such homes would appeal very strongly to many patients who would refuse to remain in the wards of a town infirmary.

